# Development and Validation of the Chinese Employee Intertemporal Decision-Making Ability Scale

**DOI:** 10.3390/bs16081349

**Published:** 2026-08-05

**Authors:** Dawei Wang, Muze Li, Xiaolong Chen, Yicheng Wang, Wenxu Mao, Cuiyu Pang, Feng Qiu, Peng Yu, Yixin Hu

**Affiliations:** 1 Faculty of Psychology, Shandong Normal University, Jinan 250358, China; 2 Interdisciplinary Research Center for Applied Psychology, Shandong Normal University, Jinan 250014, China; 3 School of Educational Science, Kashi University, Kashi 844008, China

**Keywords:** intertemporal decision-making ability, employee, scale development, psychological processing capacity

## Abstract

**Objective:** Intertemporal decision-making ability refers to an individual’s capacity to evaluate and integrate temporally distributed outcomes, regulate goal-directed behavior, and construct future representations in intertemporal choice contexts. This construct reflects a core psychological processing capacity rather than a mere preference for specific choice outcomes. It plays a critical role in employees’ long-term performance management, career development, and self-regulation. However, existing research lacks standardized measurement instruments that directly assess intertemporal decision-making ability among employee populations. Therefore, the present study aimed to develop and validate a structurally clear, reliable, and valid scale of employees’ intertemporal decision-making ability applicable to Chinese organizational contexts. **Methods:** Using a sample of full-time employees from China, this study developed the scale based on a three-process framework of intertemporal decision making, encompassing three core psychological mechanisms: cognitive control, value evaluation, and future representation. Exploratory factor analysis (*N* = 433) and confirmatory factor analysis (*N* = 281) were conducted to examine the scale’s factor structure. Impulsivity and job autonomy were employed as criterion variables to assess criterion-related validity. **Results:** The findings supported a three-factor structure comprising career executive control, long-term career reward trade-off, and career future representation. The final scale included 14 items. The Employee Intertemporal Decision-Making Ability Scale demonstrated good internal consistency and stable structural validity at both the overall and dimensional levels. In addition, intertemporal decision-making ability was significantly negatively correlated with impulsivity and significantly positively correlated with job autonomy, providing support for criterion-related validity. **Conclusions:** The Employee Intertemporal Decision-Making Ability Scale developed in this study demonstrates satisfactory reliability and validity and can serve as an instrument for assessing employees’ intertemporal decision-making ability.

## 1. Introduction

Intertemporal decision-making ability (IDMA) is conceptualized as a multicomponent psychological capacity that enables individuals to evaluate and integrate outcomes across time, regulate behavior in line with temporally extended goals, and construct cognitively elaborated representations of future states in intertemporal choice contexts (Kable & Glimcher, 2007; Peters & Büchel, 2011). Contemporary research increasingly suggests that intertemporal decision making arises from the coordination of multiple psychological processes, including cognitive control, value evaluation, and future representation (Peters & Büchel, 2011; Wang et al., 2024). Accordingly, IDMA should be understood as an integrative capacity that supports adaptive decision making across temporal horizons, rather than merely as a unidimensional tendency to favor delayed rewards.

Particularly in today’s volatile, uncertain, complex, and ambiguous (VUCA) business environment, IDMA has become increasingly important in organizational contexts (Millar et al., 2018). On the one hand, rapid technological change, intensified competition, and dynamic task demands require employees to respond quickly to immediate pressures and short-term performance expectations (Huhtala et al., 2021). At the same time, sustainable organizational performance depends heavily on employees’ long-term investments in skill development, relationship building, strategic learning, and career planning (Sung & Choi, 2013). As a result, employees are frequently required to allocate limited psychological and behavioral resources—including time, effort, attention, and learning investments—between competing short-term and long-term demands.

Such decisions rarely involve isolated choices between immediate and delayed rewards. Rather, they require the continuous integration of temporally distributed information, regulation of goal-directed behavior under interference, and construction of actionable representations of future career trajectories. Based on this perspective, this study introduces the concept of E-IDMA. It defines it as the psychological function that enables employees, in complex organizational work settings, to integrate information across temporal dimensions, maintain goal-oriented control amid multiple competing demands, and construct actionable future representations aligned with both long-term career development and organizational goals.

Previous research has shown that differences in E-IDMA provide a key basis for interpreting the logic underlying workplace dynamics (Inceoglu et al., 2018). For example, employees who can maintain a stable balance between short-term pressures and long-term goals are more likely to sustain investment in capability development, task execution, and career planning. Such behavioral patterns are significantly associated with higher levels of work engagement, job satisfaction, proactive behavior, and stronger organizational commitment (Hernández-Toledano et al., 2025; Parker et al., 2010; De Ruijter et al., 2023; Sutcliffe et al., 2019). Consequently, developing a context-sensitive and theoretically grounded measure of E-IDMA is necessary for advancing research on employee self-regulation, career development, and sustainable performance.

To address this gap, the present study aims to develop and validate the Employee Intertemporal Decision-Making Ability Scale (E-IDMA Scale) within a regional Chinese context. Specifically, this study first clarifies the theoretical foundation and conceptual distinctiveness of E-IDMA, then develops a domain-specific measurement instrument suitable for organizational contexts, and finally examines its reliability, construct validity, and criterion-related validity. Consistent with established scale development procedures (Hinkin, 1998), the research comprises four sequential studies. Study 1 employs semi-structured interviews to qualitatively explore how intertemporal decision-making ability is expressed in employees’ work experiences and to generate an initial item pool. Study 2 conducts exploratory factor analysis (EFA) to identify the scale’s underlying structure. Study 3 uses confirmatory factor analysis (CFA) to validate the stability of the factor structure. Finally, Study 4 examines the relationships among E-IDMA, impulsivity, and job autonomy to establish criterion-related validity.

## 2. Theoretical Background

### 2.1. Conceptual Evolution and Dimensionality of E-IDMA

Building on the foregoing analysis, the present study draws on a three-process framework of intertemporal decision making as its theoretical foundation (Peters & Büchel, 2011; Wang et al., 2024). We adapted this framework to organizational contexts by proposing a three-dimensional model of E-IDMA: career executive control, long-term career reward trade-off, and career future representation. Specifically, the cognitive control process, when situated in workplace contexts, primarily manifests as individuals’ capacity to inhibit competing distractions and maintain focus on long-term objectives amid frequent work disruptions and urgent task pressures. It provides top-down volitional resources to help employees pursue such long-run targets within distracting, high-performance work environments (Peng & Feng, 2014). This dimension is conceptualized in the present study as career executive control. The value evaluation process in organizational decision making is reflected in employees’ ability to systematically weigh short-term gains against delayed career-related returns. In the present study, this process corresponds to the long-term career reward trade-off. To a certain extent, it shapes employees’ affective endorsement of long-term goals and their subjective utility judgments. It therefore serves as a motivational core that supports sustained long-term behavior (Volkow & Baler, 2015).

At the employee level, the future representation process transcends superficial future imagination and constitutes an integrated form of psychological processing. It enables individuals to form clear, actionable representations of their career development trajectories. Through vivid simulation of future scenarios, it effectively transforms abstract, distal career rewards into concrete, proximal mental representations. In doing so, it enhances the subjective realism of delayed rewards and mitigates temporal discounting (Chen et al., 2019). This dimension is accordingly defined as career future representation.

Furthermore, these three processes do not operate in isolation; instead, they exhibit dynamic coordination and compensatory relationships (Ferrari & Díaz-Morales, 2007). For example, when the immediate returns of a long-term project are relatively low (i.e., weak reward trade-off), a high level of future representation can enhance the subjective value of distal rewards by constructing clear promotion-related visions and improving expectations regarding rewards and efficacy. This process, in turn, reduces the burden on cognitive control and helps individuals sustain more stable effort and persistence over time (Cona et al., 2023). Such a mechanism-based understanding enables organizations to more precisely identify employees’ “bottlenecks” in intertemporal decision-making processes. Specifically, suboptimal intertemporal performance can be traced to three distinct cognitive deficits: deficient prospection (insufficient future vision), imbalanced valuation (misalignment with organizational incentives), and control failure (difficulty resisting short-term temptations). This perspective may offer corresponding theoretical underpinnings for subsequent differentiated, future-oriented interventions and precision management for employees. Taken together, the present study proposes that E-IDMA comprises three dimensions: career executive control, long-term career reward trade-off, and career future representation.

### 2.2. Conceptual Differentiation of E-IDMA from Established Constructs

To further establish the conceptual distinctiveness of E-IDMA, it is necessary to differentiate it from several established constructs related to intertemporal decision making. Two dominant, established constructs—delay discounting and future time perspective—capture important but fundamentally distinct aspects of temporal decision making. Delay discounting describes preferences between smaller-sooner and larger-later rewards (Frederick et al., 2002). Although research on this construct yields precise behavioral indices, it primarily reflects static choice outcomes. It is therefore less sensitive to the psychological processes that sustain intertemporal decision making in complex real-world environments (Lempert & Phelps, 2016). In organizational settings, employees’ decisions are rarely reducible to one-off reward trade-offs; instead, they require continuous effort regulation, coordination of multiple goals, and adaptation to dynamically shifting constraints. Future time perspective (FTP), in contrast, conceptualizes temporal orientation as a relatively stable dispositional trait. It captures the extent to which individuals attend to and prioritize future consequences (Keough et al., 1999; Kooij et al., 2018). This construct characterizes a general time-related disposition rather than the psychological capacity to translate future concerns into tangible, effective behavior. By contrast, E-IDMA centers on the underlying psychological processes through which individuals evaluate temporally separated outcomes, manage competing demands, and form concrete, actionable future mental representations.

Beyond delay discounting and future time perspective, E-IDMA also shares partial conceptual overlap with several broader psychological and career-related constructs, including conscientiousness, career adaptability, and proactive career behavior. However, these constructs differ from E-IDMA in their theoretical focus and level of analysis. Specifically, conscientiousness is a broad personality trait reflecting tendencies toward self-discipline, responsibility, and persistence (Costa & McCrae, 1992). In contrast, E-IDMA captures a domain-specific psychological processing capacity underlying temporal decision making in organizational contexts. Similarly, career adaptability reflects psychosocial resources that facilitate coping with career transitions and vocational tasks (Savickas & Porfeli, 2012). In contrast, E-IDMA focuses specifically on the cognitive-motivational processes involved in balancing short-term demands and long-term career goals. Proactive career behavior, in contrast, refers to self-initiated behaviors enacted to shape career development (Strauss et al., 2012). Thus, it may be viewed as a potential behavioral outcome of high E-IDMA rather than as a psychologically equivalent construct. Taken together, although these constructs may correlate with E-IDMA, they differ substantially in conceptual scope, functional role, and explanatory level. Table 1 provides a systematic comparison between E-IDMA and related constructs.

### 2.3. Need for a Domain-Specific Scale

Although existing approaches provide important theoretical insights, their applicability to organizational contexts remains limited. It is worth noting that Wang et al. (2024) developed a scale measuring adolescents’ intertemporal decision-making ability based on a three-dimensional theoretical framework. The development of this scale addresses the methodological shortcoming of previous studies, which “emphasized outcomes over mechanisms” (Bulley et al., 2022). However, fundamental differences exist between adolescent and organizational contexts in decision-task characteristics and consequence structures. These differences make the adolescent-focused scale difficult to apply directly to employee populations. Specifically, with respect to task attributes, adolescents’ intertemporal decisions typically revolve around immediate temptations in academic and everyday life (e.g., choosing between entertainment and studying) and primarily emphasize impulse inhibition.

In contrast, employees’ intertemporal decisions are deeply embedded in career development, role responsibilities, and the allocation of organizational resources. They are accompanied by greater uncertainty, more severe consequences, and the involvement of multiple stakeholders’ interests. Regarding the consequences of decisions, adolescents’ choices mainly affect themselves. In contrast, employees’ decisions may be directly linked to team performance and organizational interests. Consequently, employee decision making often entails greater responsibility spillover and contextual complexity (Blankenstein et al., 2018). Therefore, although the three-dimensional structural model proposed by Wang et al. (2024) is theoretically informative, it remains insufficient to systematically capture employees’ intertemporal decision-making processes in complex organizational contexts characterized by multiple distractions, heightened responsibility, and a strong long-term orientation. Addressing this gap requires moving beyond generalist measures toward a domain-specific operationalization that reflects the structural and functional demands of employee decision making.

## 3. Criterion-Related Validity and Hypothesis Development

In addition, to establish the validity of the newly developed scale, the present study examines the relationships between E-IDMA and key criterion variables. Drawing on theoretical mechanisms underlying intertemporal decision-making ability, impulsivity, and job autonomy, the present study selected impulsivity and job autonomy as criterion variables. First, impulsivity refers to a dispositional tendency to respond rapidly without adequate forethought or reflection (Fineberg et al., 2014; Van den Bos et al., 2015). Individuals high in impulsivity often lack the cognitive control required to inhibit immediate temptations and have difficulty engaging in farsighted future planning, which stands in direct contrast to the core features of intertemporal decision-making ability. Prior research has further demonstrated that higher levels of impulsivity are significantly associated with higher delay discounting rates, indicative of lower intertemporal decision-making ability (Joireman et al., 2008). Accordingly, the present study hypothesizes that E-IDMA is negatively related to impulsivity.

Second, grounded in self-determination theory, job autonomy is defined as employees’ capacity to independently carry out their work, make decisions, and choose the means to achieve their goals. It represents a psychological characteristic that reflects individuals’ active control over the work process (Hackman & Oldham, 1976; Ryan & Deci, 2000). More specifically, individuals who exhibit high levels of job autonomy necessarily possess the ability to transcend immediate situational stimuli and act in accordance with internally endorsed long-term goals (Gagné et al., 2022; Slemp et al., 2018).

Within intertemporal decision-making processes, the future representation dimension enables individuals to construct clear visions of self-development, thereby providing directional guidance for autonomous behavior. In contrast, the cognitive control dimension helps individuals maintain goal-directed action in the face of distractions. This prevents them from becoming passive responders to situational cues or immediate impulses. Consequently, employees with higher intertemporal decision-making ability possess sufficient psychological resources to exert control over their work. They are therefore likely to exhibit greater job autonomy at both psychological and behavioral levels (Koomen et al., 2020). Accordingly, the present study hypothesizes that E-IDMA is positively related to job autonomy.

## 4. Study 1: Qualitative Study

### 4.1. Research Objective

The purpose of this study is to systematically examine how employees experience and express intertemporal decision-making ability in practical work scenarios (e.g., career planning, task trade-offs, and delayed rewards) through semi-structured interviews with in-service employees. Drawing on these qualitative findings within a pre-defined theoretical framework, an initial item pool was generated that is contextually grounded in organizational settings and designed to ensure strong content validity, thereby laying a solid foundation for subsequent EFA.

### 4.2. Participants

Purposive sampling was used to recruit full-time employees from a medium-sized enterprise in Shandong Province, China. This organization provides a representative context because its diverse operational, administrative, and technical roles frequently entail intertemporal trade-offs in the workplace. Inclusion criteria required full-time employment, a minimum tenure of six months, and the ability to articulate experiences in workplace decision making.

Following Francis et al.’s (2010) saturation criteria, thematic redundancy was monitored throughout the analysis. By the 18th interview, no novel codes or conceptual themes emerged regarding the psychological mechanisms of intertemporal decision making; two additional interviews confirmed data saturation. The final sample comprised 20 participants (Table 2), with diversity in gender, age, tenure, job type, and educational background, ensuring sufficient heterogeneity of interview data.

### 4.3. Interview Syllabus

The semi-structured interview protocol was developed and evaluated by experts based on a three-process framework of intertemporal decision making—cognitive control, value evaluation, and future representation—while integrating their functional meanings within organizational contexts. The protocol covered the following domains: (1) the conceptual meaning of intertemporal decision-making ability; (2) types of intertemporal decision-making behaviors exhibited by employees; (3) intertemporal decisions made by employees in their work and daily lives and the underlying decision motives; (4) factors influencing intertemporal decision-making ability; and (5) characteristics of employees with relatively high intertemporal decision-making ability. An example interview question was: “Reflecting on your work experience, have you ever made any decisions oriented toward long-term outcomes? Please describe one or two such decisions and explain the reasons behind them.”

### 4.4. Procedure and Data Analysis

Semi-structured interviews (averaging 30 min) were audio-recorded, fully transcribed, and analyzed via NVivo 11 using directed content analysis (Hsieh & Shannon, 2005). The theoretical dimensions of intertemporal decision making (cognitive control, value evaluation, and future representation) served as an a priori deductive template, complemented by inductive coding for emergent workplace behaviors. To establish inter-coder reliability, two independent researchers double-coded a random subset of seven transcripts (35% of the sample). The remaining transcripts were coded by the primary researcher after achieving satisfactory inter-rater consensus.

After three-level coding, we converted interview transcripts into scale items following standard procedures: extracting semantic units, unifying expressions, merging repetitive content, and polishing wording. Illustrative examples of the three-level coding process are presented in Table 3. Five basic rules were adopted for item construction: items should be written in first-person declarative sentences, avoid colloquial language, convey a single meaning per item, be confined to workplace scenarios, and be expressed in neutral, easy-to-read language.

### 4.5. Results

The open coding process yielded 315 initial codes, which were consolidated into 10 axial codes and further integrated into three core categories: career executive control, long-term career reward trade-off, and career future representation (see Table 4). Representative statements included “I can complete tasks in accordance with the plans I have formulated,” “Before making decisions, I carefully consider potential gains and losses,” and “I have long-term career plans.”

Inter-coder reliability was acceptable, with all Kappa coefficients exceeding 0.40 (Table 4), indicating moderate to substantial agreement according to Landis and Koch (1977). The “Importance Evaluation” node yielded a relatively low Kappa coefficient (0.51), likely due to the greater conceptual abstraction of assessing subjective career value compared with more concrete behavioral descriptions. To address this issue, items derived from this node underwent two additional rounds of expert review to ensure conceptual clarity and consistency in responses.

Based on the final coding structure, a 24-item preliminary pool was generated, comprising 9 items for Career Executive Control, 8 for Long-term Career Reward Trade-off, and 7 for Career Future Representation (see Table A1 for the full item pool). This pool was subsequently subjected to content validity and psychometric evaluation.

### 4.6. Summary

Following the coding and item generation process, a seven-member expert panel was invited to evaluate the preliminary item pool, including four academic experts in management psychology and decision making and three frontline industry practitioners. The academic experts evaluated the construct definition, theoretical framework, and dimensional structure. In contrast, the practitioners assessed the practical relevance and contextual fit of the items in workplace settings. All experts were provided with the theoretical definition of E-IDMA, the three-dimensional conceptual framework, and the full set of preliminary items.

Content validity was assessed along two criteria: relevance and necessity. First, relevance was assessed using a 4-point scale (1 = not relevant, 4 = highly relevant), from which the Item-level Content Validity Index (I-CVI) and Scale-level Content Validity Index (S-CVI) were calculated. Second, necessity was rated on a 3-point scale (1 = not necessary, 3 = necessary), and the Content Validity Ratio (CVR) was computed following Lawshe’s (1975) formula. Experts also provided qualitative feedback regarding conceptual overlap, potential response bias, and contextual suitability.

The initial evaluation demonstrated strong overall validity (S-CVI/Ave = 0.94, S-CVI/UA = 0.79). However, three items failed to meet the psychometric thresholds (I-CVI ≥ 0.78): T9 (I-CVI = 0.71, CVR = −0.14), T16 (I-CVI = 0.43, CVR = −0.43), and T20 (I-CVI = 0.71, CVR = −0.14). Consequently, these items were deleted, reducing the pool to 21 items.

Because all original items were generated in Chinese from interview transcripts, the scale items were translated into English using the translation–back-translation procedure. The final preliminary scale comprised three dimensions: Career Executive Control (8 items), Long-term Career Reward Trade-off (7 items), and Career Future Representation (6 items), utilizing a 5-point Likert framework.

## 5. Study 2: Exploratory Factor Analysis of the E-IDMA Scale

### 5.1. Participants

A total of 472 employees from a company in Shandong Province participated in the study. Of these, 39 participants were excluded due to excessive missing data, patterned responding, or failure to pass attention check items (e.g., “Please select ‘3 = Neutral’ for this item”). The final sample comprised 433 employees (222 men and 211 women), with an effective response rate of 91.73%. Their ages ranged from 22 to 60 years (*M* = 38.88, *SD* = 8.30). Among them, 57 participants (13.16%) had less than 5 years of work experience, 173 (39.95%) had 5 to 10 years, and 203 (46.88%) had more than 10 years. The study was approved by the Academic Committee of Shandong Normal University and in accordance with the 1964 Declaration of Helsinki.

### 5.2. Measures

The initial E-IDMA consisted of 21 items (e.g., “I can complete my work efficiently without procrastinating”), rated on a 5-point Likert scale (1 = Strongly Disagree, 5 = Strongly Agree). An attention-check item (“Please select ‘3 = Neutral’ for this item”) was inserted between Items 5 and 6, and only participants selecting the correct response were retained. Cronbach’s *α* for the initial scale was 0.75.

### 5.3. Procedure and Data Analysis

Data were collected through the Credamo online survey platform with organizational assistance. Participation was voluntary, anonymous, and compensated upon completion. All participants provided informed consent. Data were analyzed using SPSS 26.0 and Mplus 8.3.

### 5.4. Item Screening and Exploratory Factor Analysis

In accordance with the extreme group criterion proposed by Kelley (1939), the top 27% of participants were categorized as the high-score group, and the bottom 27% as the low-score group. An independent-samples *t*-test revealed significant differences between the two groups (*t* = −37.07, *p* < 0.001, Cohen’s *d* = 4.85), indicating satisfactory item discrimination. Subsequently, corrected item-total correlation (CITC) analysis was conducted (see Table 5). Seven items (T4, T5, T7, T8, T9, T20, and T21) showed CITC values below the 0.30 criterion and were flagged for further evaluation (Field, 2013; George & Mallery, 2019).

Principal Axis Factoring (PAF) with Direct Oblimin rotation was conducted to examine the factor structure of the E-IDMA Scale (see Table 6). Bartlett’s Test of Sphericity was significant, *χ*^2^(210) = 3339.69, *p* < 0.001, and the Kaiser–Meyer–Olkin (KMO) value was 0.92, indicating excellent sampling adequacy for factor analysis. The pre-specified item exclusion criteria were as follows: (a) items with CITCs below 0.30; (b) items with communalities below 0.30; (c) items with primary factor loadings below 0.30, or a difference of less than 0.20 between the highest loading and the largest cross-loading; and (d) items whose highest loading did not align with the theoretically hypothesized dimension.

Parallel analysis and scree plot inspection both supported a three-factor solution. The first three empirical eigenvalues (6.694, 1.551, 1.440) exceeded the corresponding random eigenvalue (1.229), whereas the fourth did not. These results suggested that retaining three latent factors was the optimal solution.

Following a comprehensive evaluation against the above criteria, seven items (T4, T5, T7, T8, T9, T20, and T21) were removed from the item pool.

EFA was then rerun on the remaining 14 items for cross-validation (see Table 7). Parallel analysis again supported a three-factor solution. The first three empirical eigenvalues (6.665, 1.508, 1.387) were all larger than the 95% percentile random thresholds (1.378, 1.285, 1.226). In contrast, the fourth eigenvalue (0.563) was lower than the critical value of 1.171. Bartlett’s test remained significant, *χ*^2^(91) = 3225.22, *p* < 0.001, and KMO increased to 0.94, indicating excellent sampling adequacy. The revised three-factor model explained 59.61% of the total variance, supporting a stable and theoretically interpretable factor structure. Inter-factor correlations were significant and positive (*r* = 0.49–0.56, *p* < 0.001), indicating that the three dimensions were substantially related yet conceptually distinct (see Table 8).

For the revised 14-item E-IDMA Scale, the Cronbach’s *α* coefficients for the total scale, Career Executive Control, Long-Term Career Reward Trade-off, and Career Future Representation were 0.91, 0.86, 0.91, and 0.83, respectively. The three factors aligned well with the theorized dimensions of cognitive control, value evaluation, and future representation, providing empirical support for the proposed three-process framework of E-IDMA.

### 5.5. Summary

The final version of the E-IDMA Scale consists of 14 items across three dimensions: Career Executive Control (4 items), Long-Term Career Reward Trade-off (6 items), and Career Future Representation (4 items). Overall, the scale demonstrated satisfactory reliability and a stable three-factor structure.

## 6. Study 3: CFA of the E-IDMA Scale

### 6.1. Participants

A total of 305 employees from Shandong Province participated in the present study. Among them, 24 participants were excluded due to excessive unanswered items, patterned responding, or incorrect responses to attention-check items (e.g., “Please select ‘3 = Neutral’ for this item”). The final sample consisted of 281 employees (178 men and 103 women), with an effective response rate of 92.13%. Their ages ranged from 22 to 59 years old (*M* = 48.29, *SD* = 7.28). Of the final sample, 33 participants (11.7%) had less than 5 years of work experience, 15 (5.4%) had 5 to 10 years, and 233 (82.9%) had more than 10 years. The study was approved by the Academic Committee of Shandong Normal University and conducted in accordance with the 1964 Declaration of Helsinki.

### 6.2. Measures

Revised E-IDMA Scale. It consists of 14 items (e.g., “I can complete my work efficiently without procrastinating”). All items were rated on a 5-point Likert scale (1 = Strongly Disagree, 5 = Strongly Agree). An attention-check item (“Please select ‘3 = Neutral’ for this item”) was inserted between Item 5 and Item 6, and only responses selecting “3” were retained as valid. In the present study, the Cronbach’s *α* coefficients for the total scale, Career Executive Control, Long-term Career Reward Trade-off, and Career Future Representation were 0.93, 0.96, 0.97, and 0.94, respectively.

### 6.3. Procedure and Data Analysis

Data were collected through the Credamo online survey platform with organizational assistance. Participation was voluntary, anonymous, and compensated upon completion. All participants provided informed consent. Descriptive statistics and reliability analyses were conducted using SPSS 26.0, whereas CFA was performed using Mplus 8.3.

### 6.4. Results

CFA was conducted to validate the three-factor structure identified in Study 2. The results indicated that the first-order three-factor model achieved adequate model fit and outperformed the alternative one-factor and two-factor competing models (see Table 9). To examine whether the three first-order dimensions could be represented by a unified higher-order construct, a second-order model was further specified and estimated, achieving satisfactory model fit (*χ*^2^/*df* = 1.69, RMSEA = 0.05, CFI = 0.99, TLI = 0.99, SRMR = 0.04). In the second-order model, the standardized loadings of Career Executive Control, Long-Term Career Reward Trade-off, and Career Future Representation on the higher-order latent construct E-IDMA were 0.63, 0.62, and 0.61, respectively (all *p* < 0.001). These findings support representing the three dimensions as indicators of a higher-order E-IDMA construct and provide empirical support for interpreting the total scale score. The statistically significant higher-order loadings indicate that the three dimensions share sufficient common variance to justify interpretation of the overall E-IDMA score, while still retaining meaningful dimension-specific variance. Accordingly, the three subscale scores may also be retained to capture dimension-specific information.

The standardized loadings of the 14 items ranged from 0.88 to 0.97, all exceeding the conservative 0.60 threshold for convergent validity, with standard errors (SEs) of 0.01–0.02. To ensure parameter robustness, 95% confidence intervals were computed for both item loadings and latent factor correlations, with all items loading significantly onto their designated factors. The composite reliability (CR) of the three dimensions ranged from 0.95 to 0.96, and the average variance extracted (AVE) ranged from 0.79 to 0.87, meeting the recommended thresholds for convergent validity. Furthermore, moderate and significant positive correlations were observed among the latent factors (*r* = 0.36–0.39, *p* < 0.001). The square root of the AVE for each dimension consistently exceeded all cross-factor correlations, supporting favorable discriminant validity. Taken together, these findings support the construct validity of the scale within the present sample.

### 6.5. Summary

The results of CFA were consistent with the original hypotheses, indicating that the 14-item E-IDMA Scale comprises three dimensions: Career Executive Control, Long-term Career Reward Trade-off, and Career Future Representation. The Cronbach’s *α* coefficients of the total scale, Career Executive Control, Long-term Career Reward Trade-off, and Career Future Representation for this 14-item scale were 0.93, 0.96, 0.97, and 0.94, respectively.

## 7. Study 4: Criterion-Related Validity of the E-IDMA Scale

### 7.1. Participants

The present study recruited 251 employees from a company in Shandong Province as its research participants. After excluding 27 participants who had excessive missing responses, exhibited patterned responding, or failed lie scale items. The final sample consisted of 224 employees (140 men and 84 women), with an effective response rate of 89.24%. Their ages ranged from 24 to 60 years old (*M* = 45.69, *SD* = 8.37). Among them, 22 participants (9.80%) had less than 5 years of work experience, 27 (12.10%) had 5 to 10 years, and 175 (78.10%) had more than 10 years. The study was approved by the Academic Committee of Shandong Normal University and conducted in accordance with the 1964 Declaration of Helsinki.

### 7.2. Measures

Revised E-IDMA Scale. It consists of 14 items (e.g., “I can complete my work efficiently without procrastinating”). The entire scale uses a 5-point Likert scale (1 = Strongly Disagree, 5 = Strongly Agree). Higher scores on the scale indicate stronger intertemporal decision-making ability. In the present study, the Cronbach’s α coefficients for the total scale, Career Executive Control, Long-term Career Reward Trade-off, and Career Future Representation were 0.94, 0.95, 0.97, and 0.89, respectively.

Brief Barratt Impulsivity Scale (BBIS). The present study used the Brief Barratt Impulsivity Scale (BBIS), developed by Morean et al. (2014) and revised by Luo et al. (2020), consisting of 8 items (e.g., “I plan tasks carefully”). The entire scale used a 4-point Likert scale (1 = Never, 4 = Often), with higher total scores indicating greater impulsivity. In the present study, the Cronbach’s α coefficient of this scale was 0.94.

Job Autonomy Scale. The present study adopted the Job Autonomy Scale, developed by Shirom (1986), which consists of 7 items (e.g., “I have freedom to decide how to do my own work “). The entire scale used a 5-point Likert scale (1 = Strongly Disagree, 5 = Strongly Agree), with higher total scores indicating greater job autonomy. In the present study, the Cronbach’s α coefficient of this scale was 0.88.

### 7.3. Procedure and Data Analysis

Data were collected through the Credamo online survey platform with organizational assistance. Participation was voluntary, anonymous, and compensated upon completion, and all participants provided informed consent. Data were analyzed using SPSS 26.0.

### 7.4. Results

The results indicated that individuals’ intertemporal decision-making ability was significantly negatively correlated with impulsivity (*r* = −0.45, *p* < 0.001) and significantly positively correlated with job autonomy (*r* = 0.18, *p* < 0.01). All three first-order dimensions were significantly negatively correlated with impulsivity (*r* = −0.35 to −0.40, *p* < 0.001). The Long-term Career Reward Trade-off dimension was significantly positively correlated with job autonomy (*r* = 0.20, *p* < 0.01). These findings demonstrate that the revised E-IDMA Scale has satisfactory criterion-related validity (see Table 10).

## 8. Discussion

### 8.1. Theoretical and Practical Implications

The present study developed and validated the E-IDMA Scale based on a three-process framework of intertemporal decision-making ability adapted to workplace contexts (Chen et al., 2019; Peters & Büchel, 2011). The findings indicate that E-IDMA demonstrates robust structural validity, strong internal consistency, and preliminary but acceptable criterion-related validity. More broadly, this study addresses the lack of context-sensitive measurement tools for intertemporal decision making in organizational settings and offers a new lens for understanding the psychological mechanisms that underlie employees’ temporally extended behavior.

Although the present study did not directly examine developmental differences, the three-dimensional structure identified here is broadly consistent with prior research in other populations, such as adolescents (Wang et al., 2024), suggesting that certain core psychological mechanisms of intertemporal decision making may generalize across contexts. However, the cross-contextual and developmental generalizability of this structure requires further examination using longitudinal and comparative designs. Traditional organizational research has often reduced long-term orientation to either a dispositional trait (e.g., future time perspective) or a behavioral tendency (e.g., procrastination; Kooij et al., 2018; Sirois, 2014; Zabelina et al., 2018). Although such constructs capture important aspects of future-oriented functioning, they offer limited insight into the psychological processes through which individuals sustain goal pursuit over time. The three-dimensional structure validated in the present study provides a more fine-grained explanation of these mechanisms (Lempert & Phelps, 2016). More importantly, these three dimensions jointly suggest that effective intertemporal decision making in organizational contexts depends not on a single self-control mechanism but on the coordinated functioning of executive regulation, temporal value integration, and future-oriented cognitive simulation.

Criterion-related analyses showed that E-IDMA was significantly negatively associated with impulsivity and positively associated with job autonomy, providing preliminary evidence for its criterion-related validity. First, the negative association with impulsivity supports the self-regulatory nature of intertemporal decision-making ability (Iodice et al., 2022). Impulsivity reflects heightened sensitivity to immediate rewards and impaired inhibitory control. In contrast, intertemporal decision-making ability relies on cognitive control and value-evaluation processes to constrain short-sighted tendencies. Second, the positive relationship between intertemporal decision-making ability and job autonomy may be interpreted from the perspective of self-determination theory. Within this framework, job autonomy is typically conceptualized as a work characteristic that reflects the degree of discretion and freedom employees experience (Hackman & Oldham, 1976; Vallerand, 2000). The present findings suggest that E-IDMA may function as an important psychological resource that supports autonomous functioning at work by enhancing employees’ capacity for self-directed behavioral regulation. At the same time, the relatively modest correlation indicates that the relationship between E-IDMA and job autonomy is likely more complex than a simple unidirectional association. On the one hand, stronger intertemporal decision-making ability may help employees make more effective use of autonomy when managing long-term goals and allocating resources.

On the other hand, highly autonomous work environments may themselves facilitate the development of employees’ intertemporal decision-making capacity by providing greater opportunities for self-directed planning and long-term decision making (Dettmers & Bredehöft, 2020). Accordingly, job autonomy may function not only as a criterion-related variable but also as a contextual antecedent or boundary condition of E-IDMA. Future research should further examine the causal direction and potential reciprocal dynamics between these constructs using longitudinal or cross-lagged designs.

Beyond these empirical findings, the study also offers important theoretical and practical implications for understanding and assessing employee intertemporal decision making in organizational contexts. The findings further clarify how E-IDMA scores should be interpreted in practice. E-IDMA scores reflect not merely future preference but the capacity to coordinate multiple intertemporal processing mechanisms. In organizational settings, employees with higher E-IDMA are more likely to sustain goal-directed effort under short-term pressures, engage in more deliberate trade-off processes when evaluating competing demands, and construct clearer and more actionable representations of future career development. These behavioral manifestations should be understood as outcomes of the underlying processing capacity rather than as defining features of the construct itself. Therefore, a high E-IDMA score reflects employees’ perceived psychological readiness and efficacy in coordinating cognitive control, value integration, and future-oriented simulation when facing intertemporal conflicts in work settings. From a practical perspective, the E-IDMA framework may offer preliminary implications for personnel assessment, talent development, and organizational intervention. Specifically, the scale may help provide initial insights into employees’ strengths and weaknesses across different intertemporal processing components. Moreover, the framework could inform the design of future interventions—such as training programs aimed at strengthening future representation or self-regulatory control—to support employees’ long-term goal pursuit in complex and dynamic work environments. The potential application of this scale in personnel assessment and leadership development also warrants further investigation, particularly in contexts requiring strategic planning, project management, or innovation-related work, where balancing immediate demands with long-term goals may be especially important.

### 8.2. Limitations and Future Directions

Despite these contributions, several limitations of the present study should be acknowledged, which also suggest important directions for future research. First, the sample was drawn exclusively from companies located in a single geographic region (Shandong Province, China), which may restrict the external validity and generalizability of the findings. Such a geographically concentrated sample may strongly reflect region-specific economic conditions, labor market characteristics, and localized organizational cultures, thereby limiting the applicability of the E-IDMA Scale to broader organizational contexts. Additionally, demographic variations across our independent samples—such as differences in participant age distributions, years of work experience, and career development stages—may account for the slight sample-specific fluctuations observed in the scale’s psychometric properties (e.g., the slightly lower inter-factor correlations in Study 3 compared to Studies 2 and 4). While these minor variations do not compromise the scale’s overall construct or discriminant validity, they highlight how sample composition might influence correlation magnitudes. Future research should therefore rigorously examine the cross-contextual generalizability and measurement invariance of the E-IDMA Scale by collecting data from diverse industries, broader geographic regions, varied occupational tenure groups, and cross-cultural contexts.

Second, the present study relied primarily on cross-sectional, single-source self-report data, which may increase the likelihood of common method bias and social desirability effects. Although procedural and statistical remedies may help alleviate these concerns, such biases cannot be fully ruled out in the current design. Moreover, the use of a cross-sectional design limits the ability to draw causal inferences regarding the relationships among E-IDMA and related constructs. Future research could adopt more rigorous methodological approaches, including behavioral experimental paradigms, multi-source assessments (e.g., combining employee self-reports with supervisor or peer evaluations), and longitudinal or time-lagged designs to capture the dynamic development and causal effects of E-IDMA over time.

Third, although the present study provided initial evidence for criterion-related validity, the criterion variables included were relatively limited. Specifically, only impulsivity and job autonomy were examined, and the association between E-IDMA and job autonomy was modest. Therefore, the current findings should be interpreted as providing only preliminary support for the scale’s external validity. Future research could include behavioral indicators (e.g., delay-discounting performance and procrastination), psychologically proximal constructs (e.g., future time perspective, self-control, and career adaptability), and externally evaluated outcomes (e.g., supervisor-rated long-term performance or proactive career behavior) as predictive criteria.

## Figures and Tables

**Table 1:** Conceptual Distinctions Between E-IDMA and Related Constructs.

Construct	Core Focus	Construct Nature	Relationship with E-IDMA
**Delay Discounting**	Preference for smaller-sooner vs. larger-later rewards	Individual decision-making preference	Reflects specific choice outcomes rather than the underlying multi-component psychological processes.
**Future Time Perspective**	Degree of future orientation and valuation	Dispositional temporal orientation	Captures generalized motivational orientation rather than domain-specific decision-making capacity.
**Conscientiousness**	Self-discipline, persistence, responsibility	Broad personality trait	Represents a stable, foundational trait; does not capture dynamic, temporal cognitive-processing mechanisms.
**Career Adaptability**	Coping resources for career transitions	Psychosocial career resource	Reflects malleable meta-competencies and resources rather than core temporal processing capacity.
**Proactive Career Behavior**	Self-initiated career-management actions	Behavioral enactment	Represents the behavioral manifestation and action-based execution, whereas E-IDMA isolates the psychological capacity driving such behaviors.
**E-IDMA**	Integration of value evaluation, cognitive control, and future representation in organizational contexts	Domain-specific Psychological processing capacity	Focal construct of the present study.

Note. E-IDMA = Employee Intertemporal Decision-Making Ability.

**Table 2:** Demographic characteristics of interview participants (*N* = 20).

Demographic Variable	Category	*n*	%
Gender	Male	9	45
	Female	11	55
Age	23–29 years	8	40
	30–39 years	7	35
	40–54 years	5	25
Working tenure	0.5–2 years	7	35
	2–5 years	8	40
	Over 5 years	5	25
Job type	Frontline staff	9	45
	Operational positions	6	30
	Management positions	5	25
Educational background	Junior college and below	6	30
	Bachelor’s degree	12	60
	Master’s degree and above	2	10

**Table 3:** Typical examples of interview content coding.

Original Interview Statement (Verbatim)	Open Codes	Axial Codes	Core Dimension
I will strictly follow the work plans I have made and finish tasks on schedule.	Follow plans; On-time execution	Work plan implementation	Career Executive Control
When making work choices, I prioritize long-term career development over temporary benefits.	Prioritize long-term development; Trade short-term gains	Long-term reward trade-off	Long-term Career Reward Trade-off
I will arrange daily work according to my long-term career goals.	Advance planning; Focus on future goals	Career future cognition	Career Future Representation

**Table 4:** Kappa Coefficients of All Nodes.

	Kappa
**Long-term Career Reward Trade-off**	
Importance Evaluation	0.51
Reward Emphasis	0.72
**Career Executive Control**	
Time Management	0.67
Delayed Reward Pursuit	0.82
Temptation Resistance	0.74
Goal-setting	0.83
Planning	0.63
Focus	0.77
**Career Future Representation**	
Future Consideration	0.82
Imagining Future Consequences	0.68

**Table 5:** Item-Total Correlations (*r_it_*) of the 21-Item Initial E-IDMA Scale(*r_it_*).

	*r_it_*
T1 I can complete my work efficiently without procrastinating.	0.64 ***
T2 I can manage my time effectively and complete my tasks on time or ahead of schedule.	0.65 ***
T3 I can complete tasks in accordance with the plans I have formulated.	0.63 ***
T4 I can control myself from engaging in activities unrelated to my current work tasks.	0.11 *
T5 When working, I can resist pleasurable distractions (e.g., using my phone or chatting).	0.16 **
T6 I am patient when working on tasks.	0.57 ***
T7 When encountering difficulties, I can think calmly.	0.16 **
T8 Even when I receive work tasks outside regular working hours, I can quickly shift into a work-focused state.	0.09
T9 I firmly believe that effort will be rewarded.	0.04
T10 I believe that working hard now will lead to better development in the future.	0.68 ***
T11 Working hard will bring me benefits or returns (e.g., social networks, salary, resources).	0.74 ***
T12 Before making decisions, I carefully consider potential gains and losses.	0.66 ***
T13 When doing things, I consider not only immediate gains but also long-term benefits.	0.73 ***
T14 To enhance my work ability, I am willing to work overtime when necessary.	0.64 ***
T15 To improve my competitiveness, I proactively learn new knowledge and actively participate in training activities.	0.69 ***
T16 When doing something, I can anticipate its potential outcomes.	0.58 ***
T17 I attach importance to the impact of my current behavior on the future.	0.55 ***
T18 When taking action, I consider possible future situations.	0.60 ***
T19 I hold positive expectations about the future.	0.63 ***
T20 I have long-term career plans.	0.12 *
T21 When doing things, I think through the consequences before taking action.	0.03

*** *p* < 0.001. ** *p* < 0.01. * *p* < 0.05.

**Table 6:** Principal Axis Factoring (PAF) for the 21-Item Scale.

Item	Career Executive Control	Long-Term Career Reward Trade-Off	Career Future Representation	Communalities
T1	0.69			0.61
T2	0.66			0.65
T3	0.74			0.65
T4		0.06		0.01
T5	0.01			0.00
T6	0.65			0.53
T7		0.06		0.00
T8	0.04			0.01
T9			0.04	0.02
T10		0.72		0.60
T11		0.76		0.71
T12		0.70		0.57
T13		0.75		0.67
T14		0.69		0.54
T15		0.74		0.62
T16			0.57	0.49
T17			0.58	0.47
T18			0.66	0.59
T19			0.69	0.65
T20		0.02		0.00
T21			0.05	0.03

**Table 7:** Principal Axis Factoring (PAF) for the 14-Item Scale.

Item	Career Executive Control	Long-Term Career Reward Trade-Off	Career Future Representation	Communalities
T1	0.76			0.61
T2	0.73			0.65
T3	0.83			0.65
T6	0.75			0.54
T10		0.77		0.60
T11		0.80		0.70
T12		0.76		0.57
T13		0.80		0.68
T14		0.75		0.53
T15		0.80		0.62
T16			0.69	0.49
T17			0.68	0.48
T18			0.77	0.59
T19			0.82	0.65

**Table 8:** Factor Correlation Matrix of the 14-Item Scale.

	Career Executive Control	Long-Term Career Reward Trade-Off	Career Future Representation
Career Executive Control	-		
Long-term Career Reward Trade-off	0.56 ***	-	
Career Future Representation	0.49 ***	0.53 ***	-

*** *p* < 0.001.

**Table 9:** Results of CFA fit indices.

Model	*χ*^2^/*df*	RMSEA	CFI	TLI	SRMR
Second-order Model	1.69	0.05	0.99	0.99	0.04
Three-factor Model (A, B, C)	1.69	0.05	0.99	0.99	0.04
Two-factor Model (B + C and A)	13.02	0.21	0.78	0.73	0.17
Two-factor Model (A + C and B)	13.01	0.21	0.78	0.73	0.17
Two-factor Model (A + B and C)	16.41	0.23	0.71	0.66	0.17
One-factor Model (A + B + C)	26.92	0.30	0.51	0.42	0.22

**Table 10:** Correlation Coefficients of E-IDMA and Its Dimensions with Impulsivity and Job Autonomy.

	*M*	*SD*	E-IDMA	Career Executive Control	Long-Term Career Reward Trade-Off	Career Future Representation	Impulsivity	Job Autonomy
**E-IDMA**	3.56	0.99	-					
**Career** **Executive Control**	3.66	1.27	0.80 ***	-				
**Long-term** **Career** **Reward Trade-off**	3.48	1.20	0.89 ***	0.53 ***	-			
**Career** **Future Representation**	3.61	1.08	0.79 ***	0.50 ***	0.57 ***	-		
**Impulsivity**	3.25	0.76	−0.45 ***	−0.35 ***	−0.40 ***	−0.38 ***	-	
**Job** **Autonomy**	4.09	0.80	0.18 **	0.11	0.20 **	0.12	0.14 *	-

*N* = 224. *** *p* < 0.001. ** *p* < 0.01. * *p* < 0.05.

**Figure A1:**
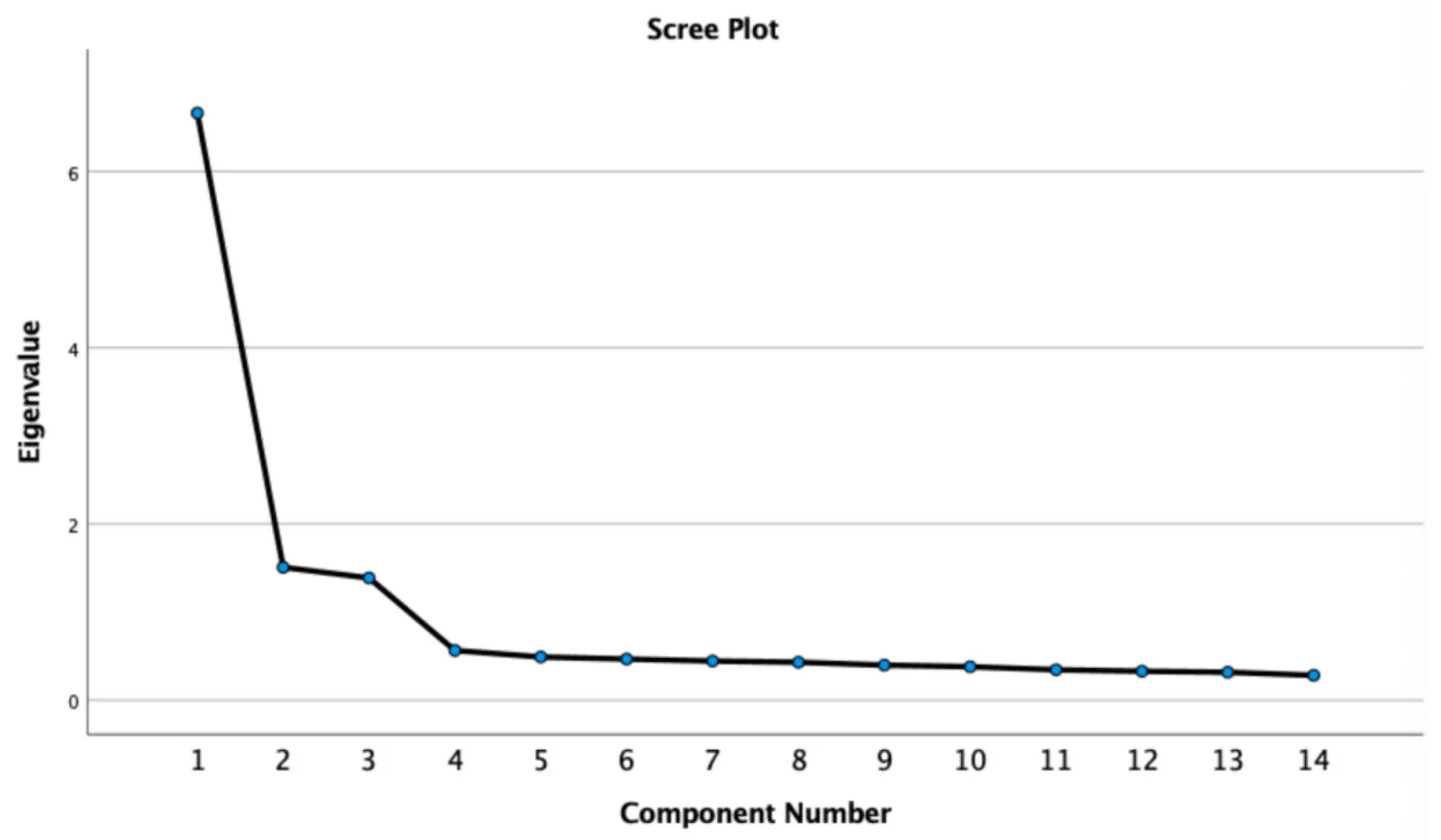
Scree Plot from the Exploratory Factor Analysis of the 14-Item Scale.

## Data Availability

The data presented in this study are open-access and can be downloaded from https://doi.org/10.5281/zenodo.21233023.

## Appendix A

**Table A1:** 24-Item Preliminary Item Pool of E-IDMA.

Item Code	Item Description
T1	I can complete my work efficiently without procrastinating.
T2	I can manage my time effectively and complete my tasks on time or ahead of schedule.
T3	I can complete tasks in accordance with the plans I have formulated.
T4	I can control myself from engaging in activities unrelated to my current work tasks.
T5	When working, I can resist pleasurable distractions (e.g., using my phone or chatting).
T6	I am patient when working on tasks.
T7	When encountering difficulties, I can think calmly.
T8	Even when I receive work tasks outside regular working hours, I can quickly shift into a work-focused state.
T9	I always maintain a positive attitude toward my work, regardless of the difficulties I encounter.
T10	I firmly believe that effort will be rewarded.
T11	I believe that working hard now will lead to better development in the future.
T12	Working hard will bring me benefits or returns (e.g., social networks, salary, resources).
T13	Before making decisions, I carefully consider potential gains and losses.
T14	When doing things, I consider not only immediate gains but also long-term benefits.
T15	To enhance my work ability, I am willing to work overtime when necessary.
T16	I believe that success mainly depends on whether I am satisfied with my current work.
T17	To improve my competitiveness, I proactively learn new knowledge and actively participate in training activities.
T18	When doing something, I can anticipate its potential outcomes.
T19	I attach importance to the impact of my current behavior on the future.
T20	I often imagine that my future life will be better than my current life.
T21	When taking action, I consider possible future situations.
T22	I hold positive expectations about the future.
T23	I have long-term career plans.
T24	When doing things, I think through the consequences before taking action.

**Table A2:** Eigenvalues and Variance Explained in Exploratory Factor Analysis (14-Item Scale) in Study 2.

Factor	Initial Eigenvalue			Extracted Eigenvalue		
	Total	Variance %	Cumulative %	Total	Variance %	Cumulative %
1	6.67	47.60	47.60	6.27	44.79	44.79
2	1.51	10.77	58.37	1.11	7.89	52.68
3	1.39	9.91	68.28	0.97	6.93	59.611

**Table A3:** Standardized Item Loadings and Standard Errors (SE) for the First-Order CFA Model in Study 3.

Item	Career Executive Control	SE	Long-Term Career Reward Trade-Off	SE	Career Future Representation	SE
T1	0.97	0.01				
T2	0.93	0.01				
T3	0.91	0.01				
T6	0.91	0.01				
T10			0.88	0.01		
T11			0.89	0.01		
T12			0.91	0.01		
T13			0.89	0.01		
T14			0.89	0.01		
T15			0.89	0.01		
T16					0.93	0.02
T17					0.88	0.02
T18					0.91	0.01
T19					0.88	0.02

**Table A4:** Composite Reliability (CR) and Average Variance Extracted (AVE) in Study 3 (CFA).

	CR	AVE
Career Executive Control	0.96	0.87
Long-term Career Reward Trade-off	0.96	0.79
Career Future Representation	0.95	0.81

**Table A5:** Factor Correlation Matrix of the Revised Employee Intertemporal Decision-Making Ability Scale in Study 3 (CFA).

	Career Executive Control	Long-Term Career Reward Trade-Off	Career Future Representation
**Career** **Executive** **Control**	-		
**Long-term** **Career** **Reward Trade-off**	**0.39 *****	**-**	
**Career** **Future** **Representation**	**0.39 *****	**0.36 *****	**-**

*** *p* < 0.001.

**Table A6:** Standardized Second-Order Factor Loadings of the Revised Employee Intertemporal Decision-Making Ability (E-IDMA) Scale in Study 3 (CFA).

First-Order Factor	Standardized Loading	SE
Career Executive Control	0.63	0.07
Long-term Career Reward Trade-off	0.62	0.07
Career Future Representation	0.61	0.07

**Table A7:** Revised Employee Intertemporal Decision-Making Ability Scale.

Item Code	Item Description
T1	I can complete my work efficiently without procrastinating.
T2	I can manage my time effectively and complete my tasks on time or ahead of schedule.
T3	I can complete tasks in accordance with the plans I have formulated.
T4	I am patient when working on tasks.
T5	I believe that working hard now will lead to better development in the future.
T6	Working hard will bring me benefits or returns (e.g., social networks, salary, resources).
T7	Before making decisions, I carefully consider potential gains and losses.
T8	When doing things, I consider not only immediate gains but also long-term benefits.
T9	To enhance my work ability, I am willing to work overtime when necessary.
T10	To improve my competitiveness, I proactively learn new knowledge and actively participate in training activities.
T11	When doing something, I can anticipate its potential outcomes.
T12	I attach importance to the impact of my current behavior on the future.
T13	When taking action, I consider possible future situations.
T14	I hold positive expectations about the future.

Note. All items use a 5-point disagree–agree response format, in which 1 strongly disagree, 2 disagree, 3 neutral, 4 agree, and 5 strongly agree.
